# Effect of mHealth With Offline Antiobesity Treatment in a Community-Based Weight Management Program: Cross-Sectional Study

**DOI:** 10.2196/13273

**Published:** 2020-01-21

**Authors:** Youngin Kim, Bumjo Oh, Hyun-Young Shin

**Affiliations:** 1 Noom Inc Seoul Republic of Korea; 2 Department of Biomedical Systems Informatics Yonsei University College of Medicine Seoul Republic of Korea; 3 Department of Family Medicine, SMG-SNU Boramae Medical Center Seoul Republic of Korea; 4 Department of Family Medicine, Myongji Hospital College of Medicine Hanyang University Goyang-Si Republic of Korea

**Keywords:** obesity, mobile apps, mobile health, weight loss

## Abstract

**Background:**

Weight loss interventions using mobile phone apps have recently shown promising results.

**Objective:**

This study aimed to analyze the short-term weight loss effect of a mobile coaching intervention when it is integrated with a local public health care center and a regional hospital’s antiobesity clinic as a multidisciplinary model.

**Methods:**

A total of 150 overweight or obese adults signed up to complete an 8-week antiobesity intervention program with human coaching through a mobile platform. Paired *t* tests and multiple linear regression analysis were used to identify the intervention factors related to weight change.

**Results:**

Among the 150 participants enrolled in this study, 112 completed the 8-week weight loss intervention. Weight (baseline: mean 77.5 kg, SD 12.9; after intervention: mean 74.8 kg, SD 12.6; mean difference −2.73 kg), body mass index, waist circumference, fat mass (baseline: mean 28.3 kg, SD 6.6; after intervention: mean 25.7 kg, SD 6.3; mean difference −2.65 kg), and fat percentage all showed a statistically significant decrease, and metabolic equivalent of task (MET) showed a statistically significant increase after intervention. In multiple linear regression analysis, age (beta=.07; *P*=.06), △MET (beta=−.0009; *P*=.10), number of articles read (beta=−.01; *P*=.04), and frequency of weight records (beta=−.05; *P*=.10; *R*^2^=0.4843) were identified as significant factors of weight change. Moreover, age (beta=.06; *P*=.03), sex (female; beta=1.16; *P*=.08), △MET (beta=−.0009; *P*<.001), and number of articles read (beta=−.02; *P*<.001; *R*^2^=0.3728) were identified as significant variables of fat mass change.

**Conclusions:**

The multidisciplinary approach, combining a mobile health (mHealth) care app by health care providers, was effective for short-term weight loss. Additional studies are needed to evaluate the efficacy of mHealth care apps in obesity treatment.

## Introduction

### Background

Obesity is one of the most prominent problems in the global public health domain. Worldwide, in 2016, more than 1.9 billion adults were overweight, and 650 million were obese [1]. The global prevalence of obesity nearly tripled between 1975 and 2016 [1]. Obesity is well known to increase the risk of high blood pressure, dyslipidemia, type 2 diabetes, and cardiovascular diseases [2]. Exercise and dietary interventions are necessary to prevent and control obesity.

Dietary restriction and increased physical activity are known treatments for obesity. Self-weighing is also known to be associated with weight loss [3]. To deliver the best weight loss outcome using lifestyle modification and weight logging, multiple approaches using mobile technology have been applied, such as the use of telephone calls [4], text messaging [5,6], and mobile apps [7,8]. Some studies have shown that providing supplementary mobile intervention can enhance conventional offline weight loss intervention based on the clinical setting [9,10]. The limitation of prior studies related to the combination of mobile and offline interventions was that the mobile component mostly focused on the monitoring aspect. We wanted to enhance the mobile intervention by integrating human coaching to the monitoring aspect and also to enhance the offline intervention by integrating a local public health center, which recruits eligible participants, and a local hospital that evaluates and motivates these participants.

### Objectives

As South Korea has a well-organized health care access, high-quality medical infrastructure, and high mobile phone penetration rate [11], it is a good environment to test our multidisciplinary model.

The purpose of this study was to analyze the short-term weight-loss effect of mobile coaching intervention when it is integrated with a local public health care center and a regional hospital’s antiobesity clinic as a multidisciplinary model. For mobile coaching intervention, we used a commercially available mobile app to target a broader population.

## Methods

### Study Design

**Figure 1:**
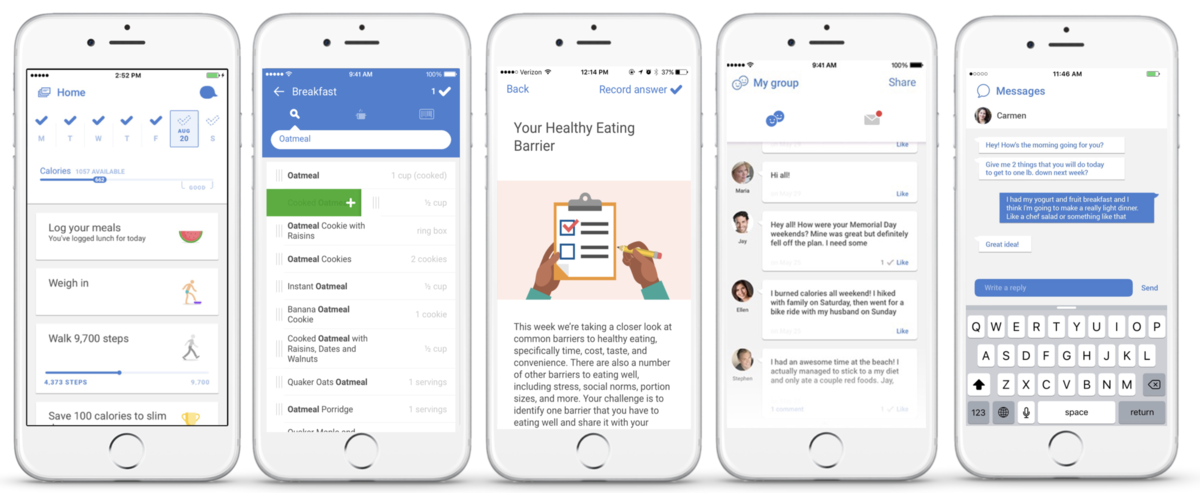
Features of the Noom Coach app.

This was a single-arm study that assessed the impact of a mobile short-term weight loss intervention combined with a conventional offline intervention based on the local health care infrastructure. Participants were recruited by a local public health care center and sent to Myongji Hospital, a local hospital, for the initial offline intervention led by a physician. An 8-week mobile intervention using the Noom Coach (Noom Inc) was provided to the participants with the offline intervention after each participant underwent an orientation of the antiobesity program (Figure 1). Various measures were assessed after the 8-week weight loss intervention.

### Recruitment

Participants were recruited by Ilsan Dong-gu Public Health Center, a local public health center run by the government. A total of 150 participants were recruited from March 2017 to September 2017. We included participants who were overweight or obese, defined as a body mass index (BMI) greater than or equal to 23 kg/m^2^ or waist circumference greater than or equal to 90 cm for men and greater than or equal to 80 cm for women per the World Health Organization criteria for Asian population [12]. Participants who showed no willingness to lose weight or did not have a mobile phone or could not visit both public health centers and hospitals were excluded. We also excluded pregnant women and subjects diagnosed as having cardiovascular or cerebrovascular disease within the recent 6 months. All the participants provided written informed consent. The Myongji Hospital Institutional Review Board (IRB) approved the study protocol (IRB no. 2018-03-012).

### Antiobesity Program—Offline Intervention

All participants visited the hospital’s antiobesity clinic once a month during the 8-week study period. The offline weight loss intervention consisted of an evaluation of the participants’ initial obesity status followed by consultation for lifestyle modification with an obesity specialist. All the participants were recommended by physicians and dietitians to reduce 500 kcal from their daily dietary intake [13]. The participants were also asked to record their intakes using the mobile app. In addition, all the participants were encouraged to perform aerobic exercise for at least 5 hours per week [13]. During each monthly visit, the participants were re-evaluated by the health care providers, who provided feedback about their calorie intake, and were encouraged to increase or maintain a moderate exercise level.

### Antiobesity Program—Mobile Intervention

**Figure 2:**
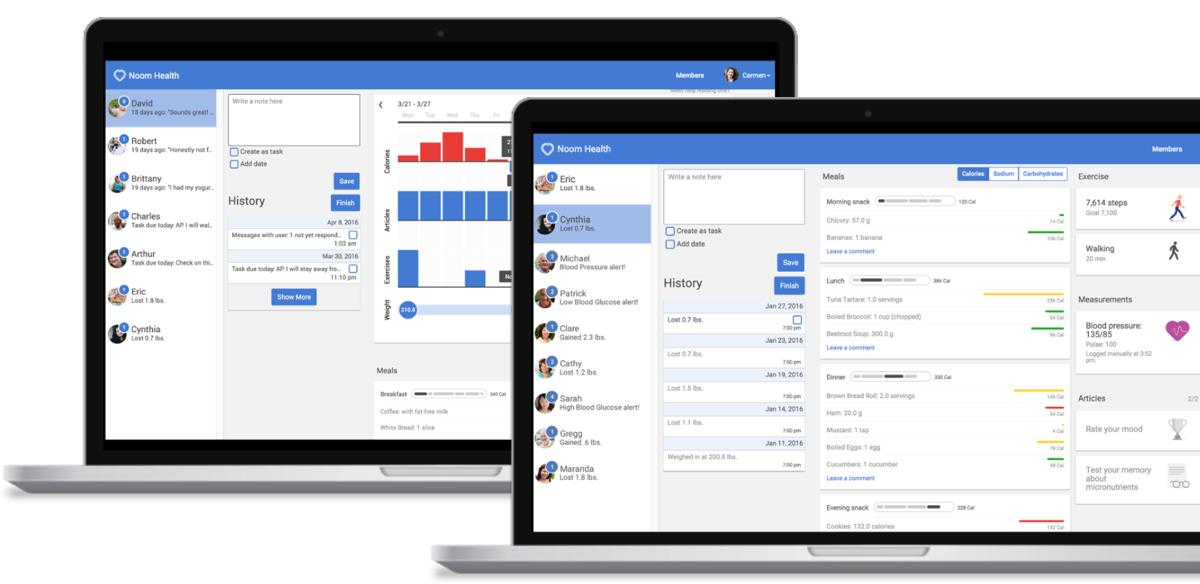
Web-based dashboard.

Noom Coach is a commercialized mobile app launched in 2012 that provides various lifestyle-related logs and is available in Android and Apple app markets. The mobile intervention consisted of (1) food logging, (2) exercise logging, (3) weight logging, (4) in-app group activities, (5) in-app articles, and (6) messages from the coaches. Total counts using mobile app is the sum of diet records, exercise records, weight records, all in-app group records, number of messages, and number of articles read. The participants were encouraged to log their food intake and exercises on a daily basis and record their weight on a weekly basis. The participants were assigned to an in-app group where they can communicate with other participants, sharing their healthy behaviors. In-app articles were also provided for the participants to gain knowledge about healthy dietary intake and physical activities. In-app articles were written by physicians, nutritionists, and clinical psychologists. Individual coaches offered feedback to the participants based on their entries with praise, emotional support, encouragement, and validation. A Web-based dashboard was used to monitor the participants’ data and provide individualized feedback (Figure 2). The coaches communicated with the participants via in-app messaging at least two times per week. The coaches and participants discussed their health-related struggles and set up realistic behavioral health goals on a weekly basis. The health goals were related to calorie restriction and increased aerobic physical activity. This process considered each participant’s habits, preferences, and resources. For example, walking 5000 steps a day using an in-app pedometer, drinking 8 cups of water a day, and logging their food intake throughout the day were identified as missions for the participants to implement; the coaches followed up to see whether the participants had completed them. If they were successful, the coaches offered praise and set another goal. If they were unsuccessful, the coaches and participants discussed the barriers and set up another realistic goal.

The coaches received education from physicians, clinical nutritionists, and clinical psychologists before starting coaching services regarding the medical, nutritional, and behavioral components of obesity and weight loss. Among the 93 techniques of the Behavior Change Technique Taxonomy [14], 10 were used during the mobile intervention. Social reward and feedback on behavior were provided by the coaches. The in-app curriculum contained content related to habit formation, graded tasks, action planning, problem solving, and goal setting. Self-monitoring of behavior was applied by letting participants record their food intake and exercise. Social support was provided by the in-app groups.

### Measurements

All measurements were taken according to a standard protocol at baseline and week 8. Adiposity indices, such as BMI, waist circumference, and body composition, were measured by registered nurses at both public health centers and the Myongji Hospital. Anthropometric measurements were performed with the subjects wearing light clothing without shoes. BMI was calculated by dividing weight by height squared (kg/m^2^). Waist circumference was measured at the midpoint between the lower border of the rib cage and the iliac crest. Body composition, including whole-body muscle mass, fat mass, and fat percentage, was estimated using a bioelectrical impedance analysis device (InBody230; InBody). Blood pressure was measured in the right arm after the participant was seated and allowed to rest for 15 min; two measurements were recorded. Metabolic equivalent of task (MET) was measured to track the exercise levels of the participants. MET was calculated using the MET formula using the exercise intensity and duration and physical activity data collected during the survey [15].

### Statistical Analysis

The mean values and standard deviations of all variables before and after the weight reduction were calculated. The mean difference and 95% CI of all variables were also calculated. A paired *t* test and multiple linear regression analysis were used. Covariates with *P* values <.15 in the univariate analysis of their association with changes in weight and fat mass were further considered in the multiple linear regression model. The factors included in the final multiple regression model were selected based on statistical significance and clinical importance. For model 1, we used *total counts using a mobile app* during mobile intervention in the regression model. For model 2, we included all mobile health (mHealth) components in the regression model. We presented an effect estimate for the interquartile increase in each variable on weight, which was calculated by multiplying the regression coefficient by the interquartile range (75th–25th percentile) of each variable. The equation was applied for effective comparison of the effect estimates across various variables with different units and easy interpretation of weight change corresponding to a realistic increment of each risk factor [16]. We analyzed all data using the statistical program SAS 9.2 (SAS Institute, Cary, North Carolina). *P* values <.05 were considered significant.

## Results

### Demographic Data and Comparison of Participant’s Characteristics Before and After the Intervention

Among the 150 participants, 112 completed the 8-week intervention (completion rate 74.7%). The mean age of the participants was 47.8 years (SD 10.2), and the proportion of females was 72.3% (82/112). The values of the variables before and after the 8-week intervention are presented in Table 1. Weight, BMI, waist circumference, fat mass, and fat percentage all showed a statistically significant decrease. MET showed a statistically significant increase, whereas muscle mass did not show any significant change.

Table 2 shows the mean total use frequency and each mHealth component during the 8-week antiobesity intervention.

**Table 1:** Clinical characteristics of the study subjects and comparison of change after intervention (N=112).

Variables	Baseline	After weight reduction	Mean difference	95% CI	*P* value^a^
Age (years), mean (SD)	47.8 (10.2)	—^b^	—	—	—
Female, n (%)	82 (72.3)	—	—	—	—
Weight (kg), mean (SD)	77.5 (12.9)	74.8 (12.6)	−2.73	−3.36 to −2.41	<.001
Height (cm), mean (SD)	163.6 (8.4)	—	—	—	—
BMI (kg/m^2^), mean (SD)	28.8 (3.3)	27.8 (3.1)	−1.03	−1.27 to −0.91	<.001
Waist (cm), mean (SD)	94.2 (10.9)	90.5 (10.2)	−3.39	−4.62 to −2.76	<.001
Muscle mass, mean (SD)	27.0 (5.9)	27.2 (6.1)	0.19	−0.10 to 0.34	.21
Fat mass, mean (SD)	28.3 (6.6)	25.7 (6.3)	−2.65	−3.24 to −2.35	<.001
Fat percentage, mean (SD)	36.7 (6.0)	34.5 (6.6)	−2.22	−2.85 to −1.90	<.001
Systolic BP^c^ (mm Hg), mean (SD)	126.5 (16.9)	125.0 (14.5)	−1.19	−4.05 to 0.27	.42
Diastolic BP (mm Hg), mean (SD)	79.1 (11.5)	77.7 (10.5)	−1.73	−3.71 to −0.72	.09
Metabolic equivalent (kcal/min/kg), mean (SD)	790.1 (841.2)	1365.1 (1507.4)	504.9	264.2 to 622.6	<.001

^a^Paired *t* test.

^b^No change from baseline value.

^c^BP: blood pressure.

**Table 2:** The mean counts of participants’ mHealth components during 8 weeks of intervention.

mHealth components	Number of use per participants, mean (SD)
Total counts using a mobile app^a^	309.5 (254.6)
Frequency of diet records	105.2 (69.8)
Frequency of exercise records	21.0 (28.9)
Frequency of weight records	9.5 (13.2)
Number of articles read	86.5 (78.2)
Number of posting on in-app group	10.7 (19.1)
Number of replies to in-app group	7.3 (17.3)
Number of expressing a favor on in-app group	14.6 (41.4)
Number of coaching communications (in-app messages)	54.8 (57.0)

^a^Total counts using a mobile app is the sum of diet records, exercise records, weight records, all in-app group records, number of messages, and number of articles read.

### Factors Associated With Weight Reduction

The variables associated with weight changes in the multiple linear regression analysis are displayed in Table 3. Age (beta=.06; *P*=.04), △MET (beta=−.0009; *P*<.001), and total counts using a mobile app (beta=−.005; *P*<.001; *R*^2^=0.3936) significantly predicted short-term weight loss. Moreover, specific components of total counts using a mobile app were analyzed with multiple linear regression. Age (beta=.07; *P*=.06), △MET (beta=−.0009; *P*=.10), number of articles read (beta=−.01; *P*=.04), and frequency of weight records (beta=−.05; *P*=.10; *R*^2^=0.4843) were identified as significant factors of weight change (Table 3). Moreover, we tried to identify variables associated with fat mass changes in the multiple linear regression analysis. As a result, age (beta=.05; *P*=.07), sex (female; beta=1.08; *P*=.10), △MET (beta=−.0009; *P<*.001), and total counts using a mobile app (beta=−.005; *P*<.001; *R*^2^=0.3559) significantly related to fat mass change. In the analysis of specific components of the total counts using a mobile app, age (beta=.06; *P*=.03), sex (female; beta=1.16; *P*=.08), △MET (beta=−.0009; *P*<.001), and number of articles read (beta=−.02; *P*<.001; *R*^2^=0.3728) were identified as significant variables. The results indicated that for every 1-year increase in age, there was a corresponding 0.07 kg increase in weight and 0.06 kg increase in fat mass. For every unit increase in MET, there was a 0.0009 kg decrease in weight and 0.00009 kg decrease in fat mass. Furthermore, for every one-time increase in the number of articles read, there was a 0.01 kg decrease in weight and a 0.02 kg decrease in fat mass, and for every one-time increase in weight record, there was a 0.05 kg decrease in weight in our study. In another explanation, the weight change per the interquartile range increase in age, △MET, and total counts using a mobile app were 0.75, −0.75, and −2.24 kg, respectively, and fat mass change per the interquartile range increase in age, △MET, and total counts using a mobile app were 0.63, −0.75, and −2.24 kg, respectively, in model 1. The fat mass change per the interquartile range increase in age, △MET, number of articles read, and frequency of weight record were 0.88, −0.75, −1.57, and −0.55 kg, respectively, and the fat mass change per the interquartile range increase in age, △MET, and total use frequency were 0.75, −0.75, and −3.14 kg, respectively, in model 2.

**Table 3:** Multiple linear regression analysis used to identify the factors provided by the antiobesity program with mHealth and clinical variables associated with weight change and fat mass change.

Variables		Weight change			Fat mass change		
		Beta coefficient	SE	*P* value	Beta coefficient	SE	*P* value
**Model 1^a,b^**							
	Age	.06	0.03	.04	.05	0.03	.07
	Sex	—^c^	—	—	1.08	0.65	.10
	ΔMET^d^	−.0009	0.0003	<.001	−.0009	0.0003	<.001
	Total counts using a mobile app^e^	−.005	0.001	<.001	−.005	0.001	<.001
**Model 2^f,g^**							
	Age	.07	0.04	.06	.06	0.03	.03
	Sex	—	—	—	1.16	0.64	.08
	ΔMET	−.0009	0.0005	.10	−.0009	0.0003	<.001
	Number of articles reads	−.01	0.006	.04	−.02	0.004	<.001
	Number of weight records	−.05	0.03	.10	—	—	—

^a^Weight change *R*^2^=0.3936.

^b^Fat mass change *R*^2^=0.3559.

^c^Not applicable.

^d^MET: metabolic equivalent.

^e^Total counts using a mobile app is the sum of diet records, exercise records, weight records, all in-app group records, number of messages, and number of articles read.

^f^Weight change *R*^2^=0.4843.

^g^Fat mass change *R*^2^=0.3728.

## Discussion

### Principal Findings

This study examined the effectiveness of the obesity management program on the basis of the hospital setting with the mobile coaching program and revealed that younger age, increased number of articles read, and the amount of exercise were significant variables for short-term weight loss.

On the basis of the analysis, younger age showed a positive correlation with weight loss effect. The accessibility of the mobile platform might have been affected by age, which was reported in other studies on mobile platforms [17-19]. The number of articles read also showed a positive correlation with weight loss effect, implying that participants who read more articles had a better result. This was also reported in other studies that used mobile apps on weight loss [20,21], indicating that strategies to increase the reading adherence to the articles provided in mobile apps will be important for mobile weight loss interventions. Furthermore, increased physical activity measured using MET showed a positive correlation with weight loss effect. The correlation between increased physical activity and weight loss outcome has been reported in many studies [22-24]. In this study, we observed a similar outcome after the mobile weight loss intervention combined with local public health centers and local hospitals.

A significant association between self-monitoring and weight loss was reported in multiple studies [25-27], and adherence to self-monitoring increased with the receipt of feedback [26]. The traditional recording method using pens and notes is cumbersome [25], and the frequency of its use decreased with time [25]. For this reason, using a mobile phone app is an alternative method because anyone can use it, regardless of the time and location [28]. Several randomized trials reported that smartphone apps help participants monitor their weight and lead to behavioral changes through actionable and personalized feedback [29,30]. Moreover, several studies showed short-term use of mobile phone app could be effective in lifestyle modification of obese people and patients with kidney disease [31-33].

One of the key factors in the mobile intervention in this study was the use of human lifestyle coaches. Although a smartphone app is an effective method for recording and self-monitoring, the general retention rate is relatively low over time for many reasons (eg, boredom of repetitive tasks, naturally decreased motivation, or simply forgetting). A mobile phone app designed for accessibility and personalized features is most important [34]. The app in our study offers personalized feedback, timely alerts for actions within 24 hours, and professional counseling by a human being, which were helpful for increasing participant engagement in the program, implementing plans, and maintaining weight loss [35]. The ideal timing, frequency, and tone of message delivery have not been sufficiently examined in the literature; therefore, these factors can be evaluated in studies in the future. Moreover, mobile weight loss intervention (combined with offline intervention) with added features to promote exercise will be strategically considered.

### Multidisciplinary Approach

**Figure 3:**
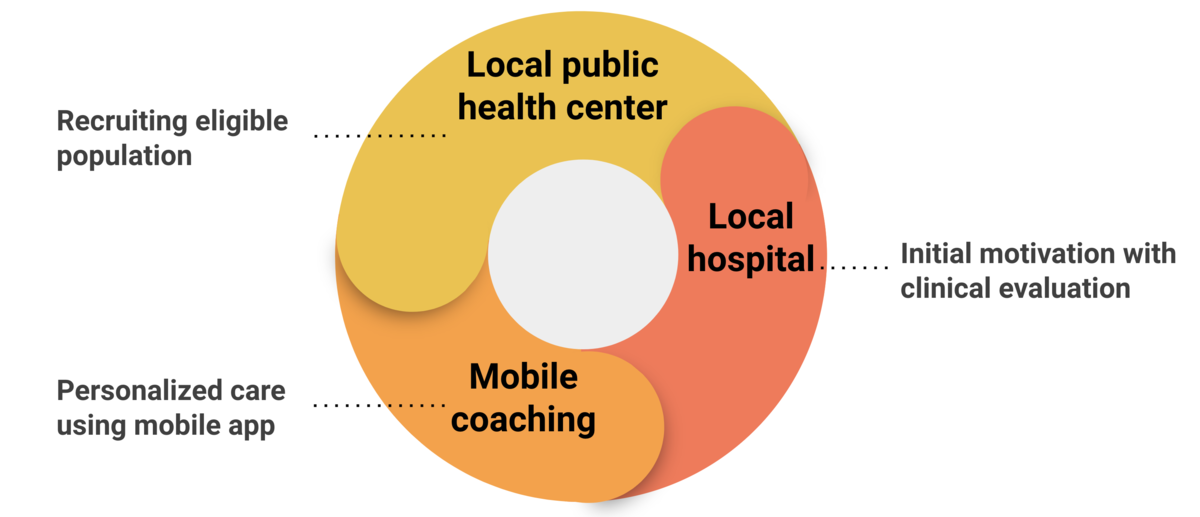
Public health, local hospital, and commercial mobile app framework.

The major strategy of our intervention was using a multidisciplinary approach to weight loss (Figure 3). Overall, three types of synergistic effects were noted during the study period. First, a synergistic effect of public and private sectors was the unique component of our multidisciplinary intervention. The combination of public health centers and private hospitals was important during the initial engagement and risk assessment. Local public health centers recruited participants with obesity on the basis of the residents’ risk data and referred them to the obesity clinics of private hospitals. Considering the difficulty encountered by private hospitals in recruiting an obese population without chronic diseases, the role of public health centers was important. Local hospitals played an important role in motivating the participants recruited by public health centers. The hospital clinic provided initial offline education along with the regular evaluation and face-to-face feedback. As there were limitations with respect to the evaluations performed and clinical expertise at the public health centers, private hospitals played an important role in providing high-quality assessment with in-depth intervention.

Second, the combination of hospitals and mobile coaching provided continuous care within the participants’ lifestyle on a daily basis. Mobile coaching supported the participants by providing them with specific daily tasks to meet the weight loss goals suggested by the hospital clinicians. The hospital clinic visits and mobile intervention supplemented their respective weaknesses. Offline encounters with and feedback from health care providers at the hospital clinic supplemented the low compliance and motivation problems of the mHealth care platform. The daily penetration of mobile coaching and easy logging supplemented the fragmented care in offline intervention.

Third, public health centers and mobile intervention showed a synergistic effect on the engagement of the users. The local public health centers promoted local resident leaderships to participate in in-app groups to better activate the mobile groups. As the group was always accessible through the mobile app, the dynamics between the participants were different from that among the participants of the offline group sessions at the local public health centers in terms of continuity.

### Limitations

Our study has several limitations. First, this was a single-arm study designed to analyze effective intervention factors related to weight change. Therefore, a randomized control trial is required to analyze the effectiveness of the intervention compared with the control group. Second, the sample size was relatively small, and the intervention duration was short, so we could not measure the sustainability and long-term effect of our intervention. Third, our study has a potential reporting bias because the mobile data collected through the mobile app were self-reported data. Under- or overreporting was possible on the basis of the characteristics of each participant. This might be from some limitation of the calibration of the frequency and types of intervention.

### Conclusions

The major outcome of this study shows that the multidisciplinary approach combining the public health sector and hospitals by health care providers with a mHealth care app had shown a short-term weight loss outcome. Further large, long-term studies are needed to evaluate the effectiveness and efficiency of the mobile health care app in an antiobesity program and to examine the possibility of a synergistic effect between mobile health care and the face-to-face approach for managing obesity.

## Abbreviations

BMI: body mass index
IRB: institutional review board
MET: metabolic equivalent
mHealth: mobile health
